# Three-Dimensional Structural Insights Have Revealed the Distinct Binding Interactions of Agonists, Partial Agonists, and Antagonists with the µ Opioid Receptor

**DOI:** 10.3390/ijms24087042

**Published:** 2023-04-11

**Authors:** Zoe Li, Jie Liu, Fan Dong, Nancy Chang, Ruili Huang, Menghang Xia, Tucker A. Patterson, Huixiao Hong

**Affiliations:** 1 National Center for Toxicological Research, US Food and Drug Administration, Jefferson, AR 72079, USA; zoe.li@fda.hhs.gov (Z.L.); jie.liu1@fda.hhs.gov (J.L.); fan.dong@fda.hhs.gov (F.D.); 2 Center for Drug Evaluation and Research, US Food and Drug Administration, Silver Spring, MD 20903, USA; nancy.chang@fda.hhs.gov; 3 National Center for Advancing Translational Sciences, National Institutes of Health, Bethesda, MD 20892, USA; ruili.huang@nih.gov (R.H.); mxia@mail.nih.gov (M.X.)

**Keywords:** µ opioid receptor, structure, binding, agonist, antagonist

## Abstract

The United States is experiencing the most profound and devastating opioid crisis in history, with the number of deaths involving opioids, including prescription and illegal opioids, continuing to climb over the past two decades. This severe public health issue is difficult to combat as opioids remain a crucial treatment for pain, and at the same time, they are also highly addictive. Opioids act on the opioid receptor, which in turn activates its downstream signaling pathway that eventually leads to an analgesic effect. Among the four types of opioid receptors, the µ subtype is primarily responsible for the analgesic cascade. This review describes available 3D structures of the µ opioid receptor in the protein data bank and provides structural insights for the binding of agonists and antagonists to the receptor. Comparative analysis on the atomic details of the binding site in these structures was conducted and distinct binding interactions for agonists, partial agonists, and antagonists were observed. The findings in this article deepen our understanding of the ligand binding activity and shed some light on the development of novel opioid analgesics which may improve the risk benefit balance of existing opioids.

## 1. Introduction

The opioid crisis is one of the most prominent and severe public health issues in U.S. history owing to its rapidly changing nature. It has affected all types of communities across the country and indeed factors including diverse geography and demography, high multiplicity and complexity of its causes, and devastating consequences from opioid misuse and opioid use disorder (OUD) have all contributed to the difficulty of combating this crisis. According to the Centers for Disease Control and Prevention (CDC) Wonder Multiple Cause of Death database (https://wonder.cdc.gov/, accessed on 13 February 2023), the number of deaths caused by opioids increased annually from 1999 to 2020. In 1999, there were 6984 opioid-related deaths, while in 2020, the number of fatalities significantly increased by approximately 14-fold to 94,371. Opioid-related deaths have been separated by the CDC Wonder database (https://wonder.cdc.gov/wonder/help/mcd.html#UCD%20-%20ICD-10%20Codes, accessed on 13 February 2023) into four specific categories: natural or semisynthetic opioids, synthetic opioids, heroin, and mental and behavioral disorders. Natural opioids such as morphine and codeine and semisynthetic opioids such as oxycodone and hydrocodone are frequently prescribed for pain reduction. Synthetic opioids such as fentanyl are often prescribed for treating severe pain, typically advanced cancer pain, although the most recent cases of harm are linked to illicitly manufactured synthetic products. Since 2013, synthetic opioids have contributed the most to the rapidly increasing trend of all opioid-related deaths and are responsible for 42% of all mortalities. The opioid crisis has not only resulted in increasing fatalities throughout the years but also financially impacts society. In November 2017, the White House suggested that previous estimations of the economic impact of the opioid crisis had been extremely underestimated in the Council of Economic Advisers’ report [1]. The annual cost of the opioid crisis increased by approximately six-fold when all losses were considered [1]. Florence et al. estimated $1.02 trillion as the economic cost of the opioid crisis, in which the number of lives lost due to opioid overdose ($480.8 billion) and the reduced quality of life due to OUD ($390.0 billion) have accounted for over 85% of the total economic burden [2]. In addition, the healthcare spending cost accounted for almost $35 billion and moreover, the criminal justice-related spending accounted for $14.8 billion [2].

Such high costs, as well as the dangers of opioid addiction, have raised public awareness of opioid misuse. Many efforts and interventions have been devoted to targeting the opioid crisis including the development of opioid prescribing guidelines; government-funded programs for OUD prevention, treatment, and recovery; and research into various aspects associated with the crisis. A recent opioid guideline was developed by CDC to explicitly address the concerns of both opioid overdose and chronic pain (https://www.cdc.gov/mmwr/volumes/71/rr/rr7103a1.htm, accessed on 14 February 2023). Government programs such as the Opioid Abuse and Overdose Prevention program of the CDC and the State Targeted Response to the Opioid Abuse Crisis program of the Substance Abuse and Mental Health Services Administration (SAMHSA) have received increased funding in response to the continuing opioid crisis. Opioid-related research has also grown, as evidenced by the surge in the number of article titles in PubMed containing the word “opioid” since 2017.

Most opioid-related research has focused on developing new drugs for pain management and OUD treatment. Unlike drugs that are only used illicitly, access to opioids cannot be overly restrictive as they remain some of the most potent painkillers in modern medicine. To reduce the potential for harm caused by opioids for pain management, alternative drugs that alleviate pain with reduced or no addiction risk are under development. Another strategy is to study the mechanism of opioid addiction and optimize current treatment regimens by combining it with other drugs and reducing doses of the opioid component. Both strategies have been investigated by researchers from a variety of perspectives and continue to be explored.

In order to elucidate the mechanisms of action of opioids, researchers have used pharmacological and biochemical approaches to investigate the signaling pathways of opioids. The experimental data were able to verify that opioids elicit their primary effects by binding to the opioid receptor, which in turn inhibits pain transmission upon activation [3,4]. Therefore, the opioid receptor plays a crucial role in the pathway involving pain transmission, making it an important drug target for pain therapy. Although opioids are highly potent painkillers, all opioids can not only cause addiction but can also manifest in dependence, tolerance, withdrawal effects, and adverse effects including respiratory depression, sedation, and constipation [4]. A major goal for researchers targeting opioid receptors is the development of new compounds that have high analgesic efficacy with fewer side effects and a lower risk of tolerance, dependence, and addiction.

The structure–activity relationship between the opioid receptor and its ligands has been extensively studied, and researchers have been able to develop derivatives of naturally occurring opioids and synthetic opioids. However, these compounds did not have much success in overcoming the adverse effects of existing opioids. With advancements in X-ray crystallography and cryogenic electron microscopy (cryo-EM), more protein structures have been resolved in recent years, including the opioid receptors. The availability of 3D protein structures enables new perspectives and allows new approaches in drug design and development, including, but not limited to, the discovery of cryptic pockets on the receptor as potential binding sites, allosteric binding sites leading to allosteric modulation of the receptor, protein–protein interaction interfaces emerging as drug targets, and polypharmacology design. Overall, 3D protein structures provide valuable insights into the development of more specific and effective treatments [5,6,7,8]. In this article, we review recently solved structures of the μ subtype of the opioid receptor. In particular, we analyze the binding site of the μ opioid receptor and ligand–receptor interactions, as well as the allosteric modulation of the μ opioid receptor.

## 2. Biology of Opioid Receptors

The hypothesis of opioid receptor existence dates back to the 1950s, and it was based on the rigid structural activity relationship of opioids [4]. The concept of selective recognition sites has led to extensive studies on the family of opioid receptors, including receptor expression, cloning, and ligand binding [9,10,11]. However, the discovery of the opioid receptor was not experimentally demonstrated until 1973 when three laboratories reported the stereoselectivity of the opioid receptor in binding assays utilizing different radioligands: [3H]naloxone [12], [3H]dihydromorphine [13], and [3H]etorphine [14]. Further investigation using pharmacologic experiments revealed the possibility of multiple subtypes of opioid receptor: μ for morphine type, δ for enkephalin type, κ for ketocyclazocine type, σ for SKF10047 (N-allylnormetazocine) type, and ε for β-endorphin type [15].

The classification of the opioid receptor subtypes has evolved as our understanding of the molecular aspects of opioid receptors has profoundly grown. After two decades of the biochemical demonstration of opioid receptors, four distinct complementary DNAs (cDNAs) were isolated and identified as members of the opioid receptor family [4]. Three of the cDNAs were later correlated to the pharmacologically defined μ-(MOR), δ-(DOR), and κ-opioid receptors (KOR), while the fourth receptor did not bind opioid ligands with high affinity [15]. Instead, a novel peptide, nociceptin/orphanin FQ was identified as the endogenous ligand for the fourth receptor in the opioid receptor family, hence the name nociceptin opioid receptor (NOR, originally named ORL-1) [16]. The amino acid sequence similarity across these four subtypes of opioid receptors is approximately 50% [15].

Opioid receptors are primarily localized in pain-modulating descending pathways, including the medulla locus coeruleus and the periaqueductal gray area [17]. They are also expressed in limbic, midbrain, and cortical structures [17,18]. Opioid receptor activation at these sites directly inhibits neurons, which leads to the inhibition of spinal cord pain transmission [17]. Meanwhile, both opioid receptors and opioid peptides can be found throughout the nociceptive neural circuitry and critical regions of the central nervous system that are involved in reward and emotion [17,18]. The endogenous opioid peptides bind to the opioid receptor with different specificity. Endomorphin-1 and -2 selectively bind to the MOR, and dynorphin-A and -B and α-neoendorphin only bind to KOR. In contrast, β-endorphin and enkephalins bind to both MOR and DOR (Table 1) [3,19,20].

In order to study the mechanism of action of the opioid receptor, elucidating its structure becomes crucial. Molecular cloning and hydropathy analysis predicted that the primary structure of the opioid receptor is composed of seven transmembrane segments, which is a structural characteristic of G protein-coupled receptors (GPCR) [15]. This prediction was validated in 2012 by X-ray crystallography [21]. The overall architecture of the opioid receptor resembles the structure of the GPCRs with seven transmembrane α-helices connected by three intracellular loops (ICL1-3) and three extracellular loops (ECL1-3) [21].

**Figure 1:**
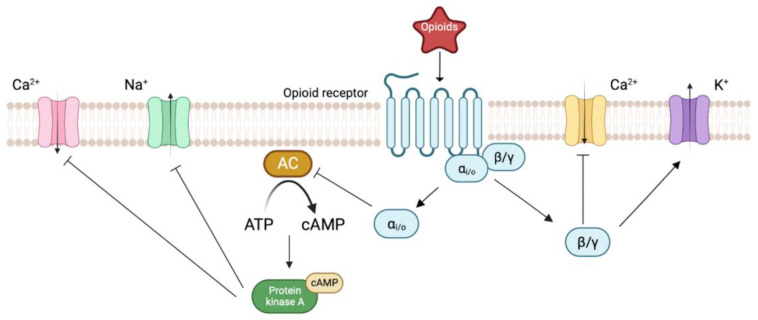
The signal transduction pathway of the opioid receptor. Upon agonist binding, the opioid receptor couples with the heterotrimeric G protein that later dissociates into Gα and Gβγ subunits, followed by the translocation of the Gα subunit that leads to the inhibition of the adenylyl cyclase (AC) activity. The release of Gβγ subunit inhibits voltage-gated Ca^2+^ channels (VGCC, L-type, and N-type) and causes activation of K^+^ channels.

As members of the non-olfactory class A GPCRs, all four known subtypes of opioid receptors are coupled to pertussis toxin-sensitive G proteins (Gi or Go or both) [17,19,20,22]. Upon agonist binding to the orthosteric binding site, the conformational change of the receptor prompts the activation of intracellular G proteins. Subsequently, the Gαi and Gβγ subunits dissociate from each other and then act on various downstream intracellular signaling pathways [17,19,20,22]. For instance, translocation of the Gα subunit is followed by the inhibition of the adenylyl cyclase (AC) activity, thereby inhibiting the formation of cAMP [23,24,25]. cAMP activates protein kinase A (PKA), a protein that is responsible for the phosphorylation of various ion channels, proteins, and enzymes, which eventually leads to their activation or inhibition [26]. The release of the Gβγ subunit leads to the inhibition of the voltage-gated Ca^2+^ channels (VGCC, L-type, and N-type) in addition to K^+^ channel activation (Figure 1) [17,24,27].

**Figure 2:**
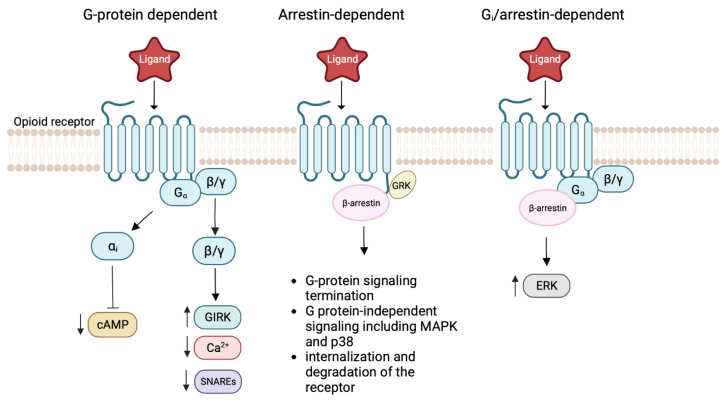
Biased signaling of MOR. The figure on the left shows the coupling between G proteins and MOR after ligand binding, leading to a reduction of cAMP, decreased Ca^2+^ signaling, and the activation of GIRK channels. In the middle, GRK phosphorylated the receptor, resulting in β-arrestin recruitment. This leads to receptor internalization and degradation. Meanwhile, β-arrestin mediates other signaling pathways, including those of MAPK and p38. The figure on the right shows the interaction between the Gαi subunit and β-arrestin. These two proteins form a complex that mediates various signaling pathways such as the activation of ERK.

GPCRs are known for their sophisticated signaling pathways and conformational landscapes. Ligands may preferentially activate or inhibit a particular pathway by altering the conformations of the receptor, and this is referred to as functional selectivity or ligand bias [28,29]. The functional selectivity of the opioid receptor has been studied extensively due to potential therapeutic benefits of biased agonism and partial agonism. The opioid receptor can have G protein dependent signaling, β-arrestin dependent signaling, and G protein β-arrestin complex dependent signaling [30,31,32]. The G protein dependent signaling leads to a reduction of cAMP, a decreased Ca^2+^ response, and the activation of G. protein-coupled inwardly rectifying potassium (GIRK) channels [33]. In contrast, the phosphorylation of opioid receptors by G protein-coupled receptor kinases (GRK) leads to the recruitment of β-arrestin [34]. The binding of β-arrestin, in turn, regulates receptor desensitization, sequestration, sorting, internalization, and degradation [34]. In this case, the β-arrestin dependent signaling terminates the G protein signaling as the opioid receptor undergoes internalization and degradation [35]. Phosphorylated β-arrestin opioid receptor complex can also recruit other signal transduction cascades such as the mitogen-activated protein kinase (MAPK) pathway and the p38 pathway [36,37]. Moreover, the formation of the Gαi subunit and the β-arrestin complex also mediate various signaling pathways, including the activation of extracellular signal-regulated kinase (ERK) (Figure 2) [37].

## 3. Structures of μ Opioid Receptor (MOR)

Several structures of MOR with atomic resolution have been reported since 2012 and are listed in Table 2.

These include two structures in complex with antagonists β-funaltrexamine (β-FNA) (PDB ID: 4DKL) [21] and alvimopan (PDB ID: 7UL4) [38]; five structures in complex with partial agonists PZM21 (PDB ID: 7SBF, 8EFO) [39,40], FH210 (PDB ID: 7SCG) [39], oliceridine (TRV130) (PDB ID: 8EFB) [40], and SR17018 (PDB ID: 8EFL) [40]; and six structures bound with full agonists BU72 (PDB ID: 5C1M) [41], [D-Ala2, N-MePhe4, Gly-ol5] enkephalin (DAMGO, a synthetic peptide) (PDB ID: 6DDE, 6DDF, and 8EFQ) [40,42], fentanyl (PDB ID: 8EF5) [40], morphine (PDB ID:8EF6) [40], lofentanil (PDB ID: 7T2H) [43], and mitragynine pseudoindoxyl (PDB ID: 7T2G) [43]. Another recently solved structure is bound with a bitopic ligand (PDB ID: 7U2L) [8]. The two structures reported earlier in 2012 [21] and 2015 [41] utilized an X-ray diffraction method and the stabilization of the receptor alone was difficult, therefore the T4 lysozyme (T4L) fusion protein strategy and nanobody were used to help stabilize the protein for crystallization. The remaining structures were from cryo-EM experiments and the receptors were solved in complex with the G proteins and/or megabodies (Table 2).

**Figure 3:**
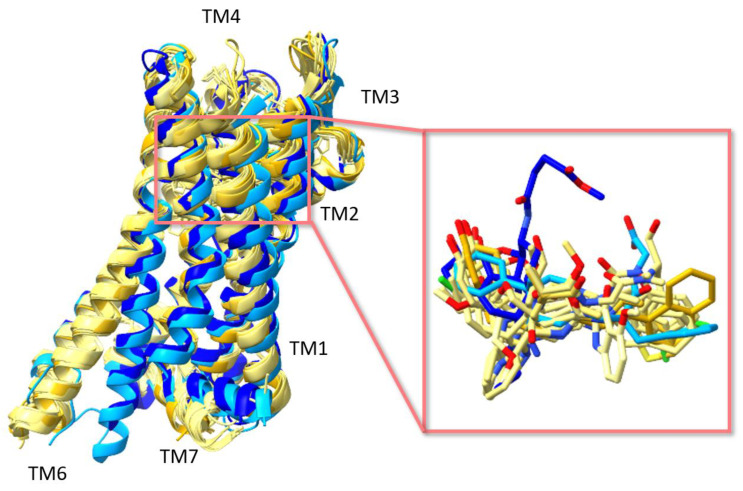
Alignment of eight MOR structures: the two MOR structures in a complex with an antagonist are in different shades of blue and six MOR structures in a complex with an agonist are in different shades of yellow. The right panel is an enlarged illustration of all ligands (both agonist and antagonist) aligned. This figure was generated with ChimeraX 1.4. [44,45].

The MOR consists of seven transmembrane helices that are connected by loops ECL1-3 and ICL1-3. The orthosteric binding site is located near the extracellular side of the helix bundle. The alignment of the structures of MOR revealed that all ligands were bound within the same site (Figure 3). Intriguingly, this binding site is largely exposed to the extracellular surface, whereas in other GPCRs the ligands were often buried deeper within the helix bundle. This structural characteristic may explain the rapid dissociation kinetics of extremely potent opioids. For instance, the inhibition constant of alvimopan is 350 pM with a half-life of 30 min [21].

**Figure 4:**
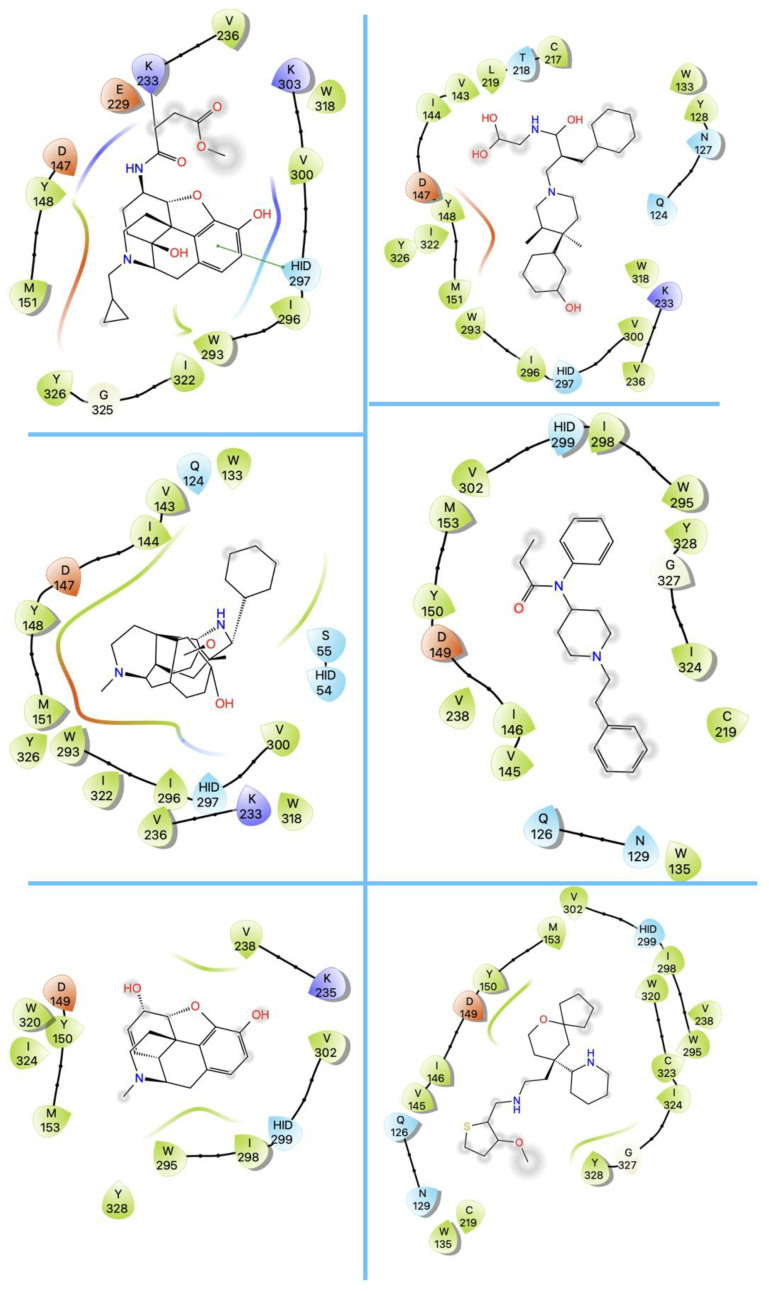

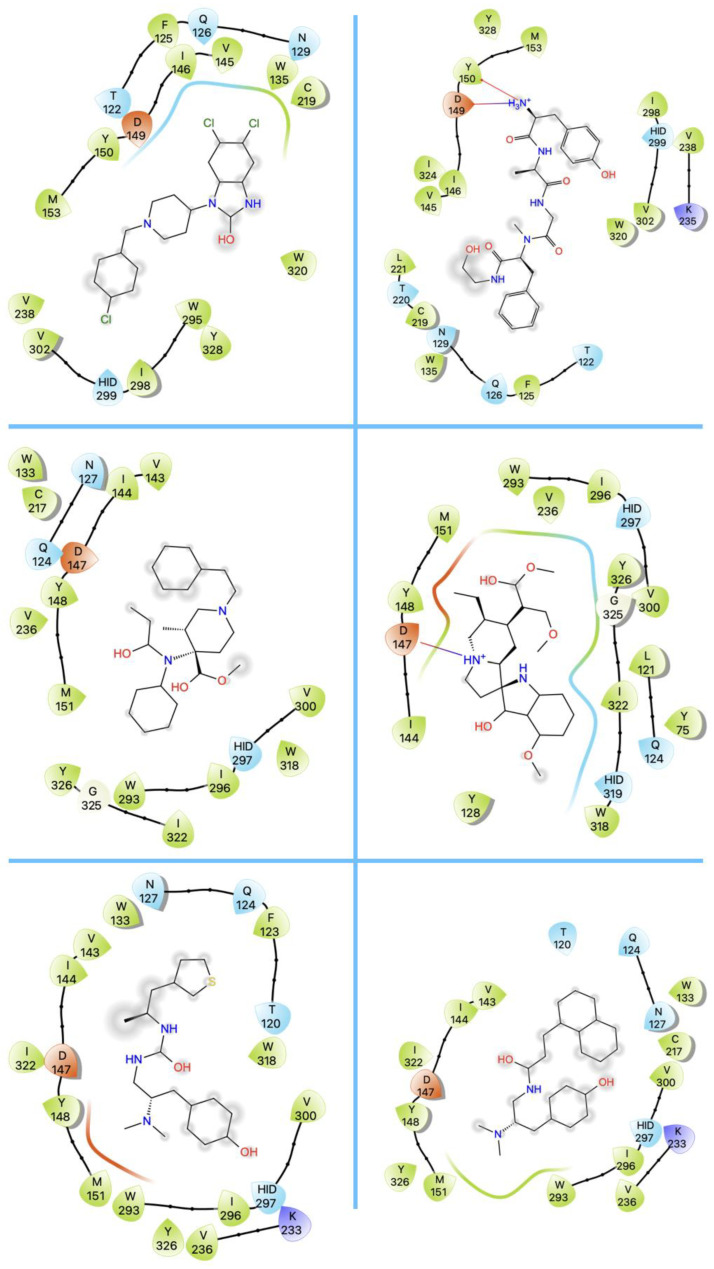
Binding pose comparison of antagonists β-FNA (**top row left**) and alvimopan (**top row right**), agonists BU72 (**row 2 left**), fentanyl (**row 2 right**), morphine (**row 3 left**), DAMGO (**row 4 right**), mitragynine pseudoindoxyl (**row 5 left**), and lofentanil (**row 5 right**), and partial agonists PZM21 (**bottom row left**), FH210 (**bottom row right**), oliceridine (TRV130) (**row 3 right**), and SR17018 (**row 4 left**). This figure was generated using Maestro. [46].

Both agonists and antagonists bind to the same orthosteric site of the MOR, and the structural differences presented in these structures were relatively subtle. In particular, the binding modes of the antagonist β-FNA and full agonist BU72 are very similar. Both ligands have a morphinan scaffold with a similar binding orientation (Figure 4). Two conserved interactions between the ligands and the receptor were observed: (1) a water-mediated hydrogen bond between the phenolic hydroxyl of the morphinan group and H297^6.52^ (superscripts are Ballesteros–Weinstein numbering in GPCRs, all residue numbering scheme in this manuscript refers to the mouse MOR, there are also human MOR cryo-EM structures available with different residue number scheme) and (2) an ionic interaction between the tertiary amine of the morphinan group and D147^3.32^. Meanwhile, the side chain of D147^3.32^ also forms a hydrogen bond with Y326^7.43^. Despite the structural differences in BU72 and DAMGO, the orientation of the residues that interacted with the full agonists are highly similar. The ionic interaction with D147^3.32^ was preserved as the N-terminus of DAMGO formed a salt bridge with the amine group of D147^3.32^, and the same amine group also formed a hydrogen bond with Y326^7.43^ in this structure (Figure 4). The same water-mediated hydrogen bond network between H297^6.52^ and the phenol group of DAMGO was observed in the molecular dynamics (MD) simulation [37]. Although β-FNA and alvimopan are both antagonists, unlike the morphinan scaffold of β-FNA, alvimopan possesses phenol-piperidine moieties which share similarities with the structure of fentanyl and its analogs. The protonated piperidine forms a salt bridge with the D147^3.32^ (Figure 4). The recently solved fentanyl-bound MOR structure has shown a direct ᴨ–ᴨ stacking interaction. between the benzene ring of fentanyl and the side chain of W293^6.48^ and Y326^7.43^, which is not presented in the morphine-bound MOR structure [40]. Additionally, a hydrophobic interaction between the phenylethyl moiety of fentanyl and a minor pocket located between TM2 and TM3 of the MOR is also absent in the morphine-bound MOR structure [40]. Mutations of the residues within this minor pocket including residues Q124^2.60^, W133^23.50^, and I144^3.27^ to alanine affected the potency of fentanyl more than that of morphine [40]. These atomic details revealed by cryo-EM structures may explain the higher potency of fentanyl to the receptor. The 2D structures of all ligands are shown in Table 3.

Although the binding pocket and binding interaction are highly similar between full agonist bound structures and antagonist bound structures, the partial agonist bound structures maintained the same interactions while also showing a distinctive feature. Both partial agonists PZM21 and FH210 retained the polar interactions between their basic amine and D147^3.32^ and water mediated hydrogen bond between their phenol hydroxy group and H297^6.52^ (Figure 4). Intriguingly, both partial agonists also showed high complementarity to a lipophilic vestibule formed by the extracellular surface of transmembrane helices (TM2 and TM3) and ECL1. The interactions between the thiolphenylalkyl moiety of PZM21 and naphthyl moiety of FH210 with this lipophilic vestibule may help to explain their partial agonism and the increase in G protein biased signaling over β-arrestin recruitment.

**Figure 5:**
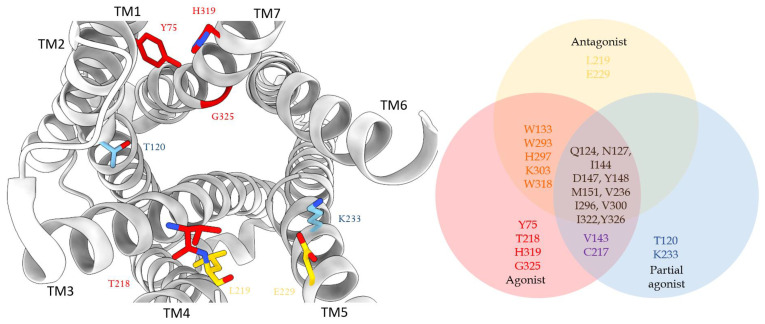
The common residues found within 4 Å for the antagonist (yellow), agonist (red), and partial agonist (light blue) are shown in licorice. The common residues found within 4 Å between the antagonist and agonist are colored orange, between the agonist and partial agonist are colored purple, and between the partial agonist and antagonist are colored green. The molecular image in this figure was generated with ChimeraX 1.4. [44,45].

Despite the overall structural similarities shared between the structures of MOR, the distinctive binding feature of the partial agonists raised the question of whether there are other differences between the binding pockets. To answer this question, we first analyzed the composition of the surrounding residues within 5 Å of the ligands in all structures. The number of residues within 5 Å of the ligands is between 15 to 20, half of which are hydrophobic, and the remaining half are either charged residues or polar residues (Table 4). This analysis suggested that the residue composition surrounding the ligands is very similar. The structure in complex with the bitopic ligand (PDB ID: 7U2L) is not included in our analyses due to the undetermined pharmacological effect of the bitopic ligand. Next, instead of focusing on the type of residues surrounding the ligands, we categorized the residues by ligand types. In other words, we investigated if the residues are shared by all ligands, between two types of ligands, or unique to one type of ligand. We also narrowed down the search by limiting the distance between the ligand and the receptor to 4 Å. A total of 11 residues were found in all structures. W133^23.50^, W293^6.48^, H297^6.52^, K303^6.58^, and W318^7.35^ were shared between structures in complex with either full agonists or antagonists. V143^3.28^ and C217^45.50^ were shared between structures in complex with either full agonists or partial agonists. Y75^1.39^, T218^45.51^, H319^7.35^, and G325^7.41^ were the residues unique to the binding of full agonists. T120^2.56^ and K233^5.39^ were the residues found within 4 Å of the partial agonist that are distinctive, and L219^5.52^ and E229^5.35^ were unique to the antagonists (Figure 5). We computed the contact area for the ligands, revealing that DAMGO has the largest contact area of 147 Å^2^ and BU72 has the smallest contact area of 40 Å^2^. As mentioned above, water molecules play an important role in mediating hydrogen bonding interactions between the receptor and the ligand. We also examined the water molecules in the structures. Out of the 15 structures, 10 structures did not solve any water molecules (PDB ID: 6DDE, 6DDF, 7SBF, 7T2G, 8EF5, 8EF6, 8EFB, 8EFL, 8EFQ, and 8EFO), while the other 5 structures (PDB ID: 4DKL, 7UL4, 5C1M, 7SCG, and 7T2H) have 2, 1, 7, 2, and 8 water molecules solved, respectively.

To further analyze the binding site, we measured the solvent-accessible surface area and solvent-accessible volume for each structure. Although residues near the ligand exhibit similar conformations, binding of the ligand may induce a conformational change in residues of the second interaction shell. Therefore, the binding of different ligands in the same binding site may still lead to subtle differences in the solvent-accessible surface area and volume of nearby residues. Table 5 lists the solvent-accessible surface area and volume of the binding site for all structures. CASTp 3.0 was used for SASA and SAV calculation [47]. There was no significant difference found in the solvent-accessible surface area among all structures, but the full agonist bound structures have the smallest solvent-accessible volume compared to that of the antagonist bound structures or partial agonist bound structures. This characteristic may be explained by the induced-fit mechanism. Although both agonists and antagonists bind to the same binding site, the size of the ligands varies, leading to differences in the shape complementarity of the binding pocket.

Molecular dynamics simulation is a technique often employed to study protein dynamics with atomic details, which provides a deeper understanding of small molecule binding and protein functional selectivity mechanisms [48,49,50,51]. Multiple studies have endeavored to investigate ligand binding and selectivity of the MOR, the role of water molecules in the MOR, the activation mechanism of the MOR, and biased signaling of the MOR using MD simulations [52,53,54,55,56,57,58,59,60,61,62,63]. Here, we summarized the findings focused on the interactions involved in ligand binding and how water molecules were involved in ligand binding to the MOR. Both Liao et al. and Podlewska et al. discovered that the partial agonist PZM21 interacts strongly with D147^3.32^, Y148^3.33^, and Y326^7.43^, which is congruent with the interactions found in the cryo-EM structures [64,65]. Intriguingly, in the MD simulation trajectories Podlewska et al. observed that the increased intensity of the interactions between PZM21 with I296^6.51^ and H297^6.52^ prompted the increased intensity of interaction with W318^7.34^ [64]. Valerylfentanyl, a fentanyl analog often implicated in opioid overdose, is a partial agonist of the MOR [66]. MD simulations have revealed that the alkyl chain of this compound is not well accommodated by the active state of the MOR, which may shift the receptor toward an inactive state [66]. Cheng et al. observed that oliceridine (TRV130) formed a hydrogen bond with D147^3.32^ and a direct interaction with Y326^7.43^, which maintained the close distance between Y326^7.43^ and W293^6.48^, thus enhancing the stability of W293^6.48^ [56]. Shim et al. showed that the salt bridge between β-FNA and D147^3.32^ was consistently maintained in MD simulations [52]. Overall, the interactions between the ligands and the MOR found in MD simulations and cryo-EM structures were consistent, while the MD simulations provided additional atomic details that may help to explain the potential mechanism of action of the partial agonism.

Fourier-transform infrared spectroscopy and UV-visible spectroscopy data have shown that internal water molecules are essential for the function of various membrane proteins including GPCRs [67,68]. It has been proposed that internal water molecules play an important role in GPCR activation and signaling, but the mechanism behind these findings remains elusive [69]. Yuan et al. performed MD simulations on the MOR to study the role of water molecules in MOR activation. Two systems were built: an agonist- (morphine) bound system and an antagonist- (levallorphan) bound system. They discovered that the agonist-bound system has more intrinsic water molecules compared to the antagonist-bound system [53]. Closer examination revealed that besides the hydrogen bond with D147^3.32^, levallorphan also formed a σ–ᴨ stacking interaction with Y320^7.43^ that prohibits water penetration [53]. These findings suggest that the size of the ligand, as well as the degree of “openness” of the binding pocket, may play a role in determining whether a ligand acts as an agonist or antagonist. Consistent with this idea, the agonist-bound structures have a smaller solvent accessible volume due to a smaller ligand size and greater exposure of the binding pocket, whereas the antagonists are larger in size, requiring the binding pocket to expand, resulting in a larger solvent accessible volume. Later, the same group published a second paper with five different systems: the apo system, agonist (morphine) bound system with and without sodium, and antagonist (naltrexone) bound system with and without sodium [55]. The analysis showed that water molecules within the binding site frequently exchange with the extracellular water molecules in the agonist-bound systems, which was not observed in the antagonist-bound systems [55]. The water molecule movement near the conserved NPxxY motifs has been associated with the switches of GPCR activation, and its importance has been demonstrated in the MD simulations [53,70,71,72]. The importance of the internal water environment near the NPxxY region has also been demonstrated by Cheng et al., in that water molecules in the agonist-bound system are much more active compared to that of the antagonist-bound system [56]. All these findings are consistent with the crystal structure data where the structure of the MOR in complex with agonist BU72 (PDB ID: 5C1M) [41] was solved with water molecules around the NPxxY region, proving the importance of water in MOR activation.

## 4. Allosteric Modulation of the MOR

With the ongoing opioid crisis, the urgency for understanding the allosteric modulation of opioid receptors to guide the development of effective and safe drugs for pain management has increased. Allosteric modulators are ligands that bind to the non-orthosteric binding site of the receptor, either enhancing (positive allosteric modulator or PAM) or diminishing (negative allosteric modulator or NAM) the signaling activities of the receptor. Allosteric modulators can alter the affinity, potency, and efficacy of the orthosteric ligands. PAMs to MOR are considered as new candidates for a safer approach to pain management. Ideally, PAMs would indirectly activate the MOR by enhancing the effects of endogenous opioid peptides, leading to the desired analgesic effect with a lower risk of severe side effects or addiction liability. Meanwhile, PAMs might increase the efficacy or potency of opioid drugs such as morphine, so the same therapeutic effect can be achieved at a lower dose with fewer side effects. It is worth noting that allosteric modulators can sometimes act as biased ligands. Bias refers to the phenomenon in which a ligand selectively activates certain signaling pathways downstream of the receptor. This phenomenon can occur for both orthosteric and allosteric ligands. In this case, the binding of PAMs may alter the preferred signaling pathway of the receptor, leading to biased downstream signaling. The advantage is the avoidance of the pathway that can potentially lead to undesired effects. For example, the β-arrestin-biased signaling of the MOR has been connected to respiratory depression and constipation [73,74,75]. Additional studies found controversy surrounding the connection between β-arrestin signaling and respiratory depression [76]. Therefore, the connection between β-arrestin-biased signaling and respiratory depression remains an area of ongoing investigation, and further studies are needed to fully understand the role of this pathway in the development of these side effects. However, PAMs may still have the potential advantage of steering the downstream signaling toward G protein-biased signaling without activating the β-arrestin-biased signaling pathway. Additionally, allosteric modulators can sometimes activate the receptor directly. Therefore, the allosteric activity is not only dependent on the binding affinity of the modulator itself and the allosteric cooperativity, which characterizes the capacity of the modulator to alter the affinity and/or efficacy of the orthosteric ligand, but also dependent on the orthosteric ligand as well [77]. The phenomenon that the same allosteric modulator has different effects depending on the orthosteric ligand is referred to as probe dependence [77]. On the other hand, two NAMs were discovered prior to the development of MOR PAMs. One of these NAMs is cannabidiol, which is a CB1 receptor agonist and was found to be a NAM of both MOR and DOR agonists [78]. Cannabidiol was shown to accelerate the dissociation of DAMGO from the MOR by a factor of at least 12 and also accelerated the dissociation of [3H]naltrindole from the DOR by a factor of at least 2 [78]. In a high-throughput screen using a β-arrestin recruitment assay, salvinorin A, a potent KOR agonist, was also identified as a NAM of MOR [79]. The potential use of NAMs may be as a treatment for opioid use disorder (OUD). As mentioned earlier, NAMs bind to the allosteric binding site of the receptor, weakening or reducing signaling activity of the receptor by slowing or inhibiting the binding of the ligands to the orthosteric binding site. In the case of the MOR, NAMs can reduce the signaling activity of the receptor, leading to a reduction in the effects of opioid agonists. This may have potential therapeutic benefits for OUD treatment. Currently, there are no MOR NAMs approved for the treatment of OUD, and more research is needed to fully explore their potential therapeutic benefits and safety profile.

**Figure 6:**
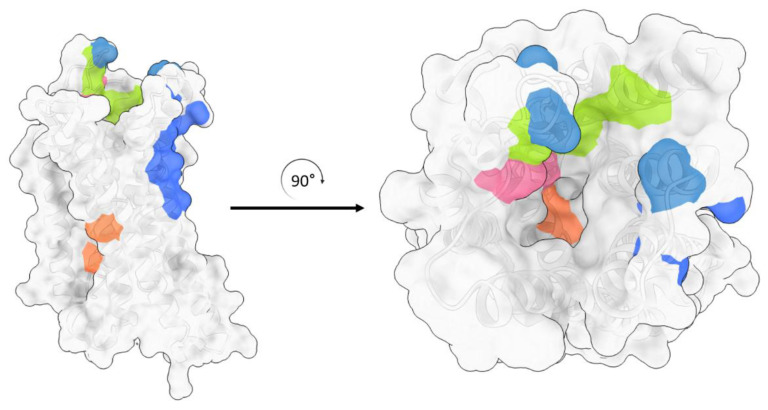
The in silico prediction of potential allosteric binding sites for PAMs and NAMs including cholesterol, small molecules, and ions. Each color depicts a potential site, and five sites are illustrated [61,80,94,97,98].

Monovalent and divalent cations, in particular the monovalent sodium cation (Na^+^) and the divalent magnesium cation (Mg^2+^), are well-known PAMs and NAMs for the MOR [61]. Interestingly, Na^+^ and Mg^2+^ have opposite effects, with Na^+^ acting as a NAM by reducing agonist binding and Mg^2+^ acting as a PAM by affecting MOR signaling [61]. The idea of Na^+^ as a NAM has been established for decades, and the hypothesis was that Na^+^ stabilizes the inactive conformation of the receptor, which later was extended to several GPCRs including the MOR [53,58]. Both high-resolution X-ray structures of GPCRs and multiple MD simulations have supported this hypothesis [58,80,81,82,83,84,85,86]. The recently solved structure of inactive DOR revealed that the allosteric binding site of Na^+^ is coordinated by two water molecules and residues N131^3.35^, S135^3.39^, and D95^2.50^ (PDB ID: 4N6H [80]) (Figure 6). Binding events of Na^+^ to this allosteric binding site were also observed in MD simulations in both inactive MOR and KOR systems [84]. This allosteric binding site was found to be collapsed in the active GPCR structures [81]. A recent study of a prototypic GPCR and adenosine A2A receptor revealed the unexpected results that Na^+^ also stabilizes the intermediate state that may correlate to partial agonism, which may explain why Na^+^ was also found to promote agonist-induced MOR activation [87]. The same study also revealed that Mg^2+^ promotes the opening of the G protein binding cleft, leading to the bridging between the acidic residues located on the extracellular region of TM5 and TM6, and resulting in receptor activation [87]. MD simulation studies have discovered that the preferred binding site of Mg^2+^ is located near the extracellular region of the MOR, in particular in proximity to residues S214^ECL2^, D216^ECL2^, and E310^ECL3^ (Figure 6) [61]. Additionally, Mg^2+^ exhibits a higher binding affinity to the active conformation of the MOR compared to the inactive conformation [61].

The conformational state of the MOR is altered not only by cations but also by other endogenous molecules such as lipids. As a membrane-embedded protein, the translocation and internalization of MOR are controlled by lipids while its function is also modulated by lipids [88], especially cholesterol, a sterol-type of lipid that comprises approximately 30% of the cell membrane [89]. Experimental data have shown that the removal of cholesterol reduces cAMP signaling of the MOR [90]. One of the ways that cholesterol can modulate the activity of the MOR is by binding directly to the receptor which leads to allosteric effects; the other is by changing the fluidity of the membrane, which in turn affects the signaling of the receptor [89]. Cholesterol was found co-crystallized with many GPCRs, including the MOR. In both active (PDB ID: 5C1M [41]) and inactive (PDB ID: 4DKL [21]) MOR structures, cholesterol was found to be co-crystallized at the same binding site. This binding site was also found in the active structures of KOR (PDB ID: 6PT2 [91], 6B73 [92]), the structure of the adenosine A2A receptor (PDB ID: 5IU4 [93]), and the active structure of the CXCR3 receptor (PDB ID: 5WB2 [94]). This binding site is a groove between TM2, TM3, and TM4 or TM6 at the extracellular region of the receptor (Figure 6).

Endogenous molecules are not the only allosteric modulators of MOR; many small molecules were either serendipitously found or intentionally designed to act as PAMs for the MOR. The first selective small molecule PAMs of the MOR are BMS-986121 and BMS-986122, both of which were identified by high-throughput screening in 2013 [95]. Both molecules were first screened for the ability to enhance the binding of endogenous agonist endomorphin-1 that recruits β-arrestin to MOR [95]. However, further studies found these compounds enhanced the affinity and/or efficacy of various opioid agonists. Consequently, more analogs of these two compounds were developed along with SAM (silent allosteric modulator) BMS-986124 [95]. PAM BMS-986122 exhibits probe dependence: it increases the potency and affinity of methadone and DAMGO but only enhanced the efficacy of morphine and nalbuphine, with no effect on the binding of antagonists [77,96]. To date, the published BMS series of compounds only act as PAMs or SAMs, and the structure–activity relationships remain unclear. However, there have been several attempts to identify the binding site of the BMS compoundseries by docking and MD simulations. Bartuzi et al. revealed that the potential binding site of BMS-986122 may be slightly above the orthosteric binding site and towards the extracellular region of TM2 and TM7 (Figure 6) [97]. Recently, Bartuzi et al. developed a novel small molecule PAM of the MOR that can enhance the efficacy of morphine [98]. They also predicted this novel compound binds in proximity to TM2 and the binding pocket is composed of W135^23.50^, C142^3.25^, V145^3.28^, I146^3.29^, and C219^45.50^ (Figure 6) [98].

## 5. Future Perspectives

Although medicinal chemists spent almost two centuries developing drugs targeting the MOR, many of the compounds still share the same scaffold as morphine or have similar synthetic scaffolds, which in turn limits the diversity of the active compounds. Moreover, this scaffold was reported to induce undesirable adverse effects, including respiratory depression and constipation. More recent approaches to developing new drugs targeting the MOR involve high-throughput screening and defined signaling assays in stable MOR transfected cell lines. However, these approaches are time-consuming and expensive. The advancement of X-ray crystallography and cryo-EM helped to solve more opioid receptor structures in recent years, which provided an unprecedented opportunity in computer-aided drug design. High-resolution structures of the MOR in complex with agonists or antagonists can confirm the binding site of the ligands and provide insights into the chemical recognition of the ligands. The molecular details of the binding site itself provide valuable information for structure-based drug design. Furthermore, some of the MOR structures have been crystallized with G proteins, which not only provides hints regarding the conformational changes that drive the downstream signaling pathway, but also in terms of the atomic details of the protein–protein interaction interface, which can potentially serve as a binding site for protein–protein interaction disruptors or molecular glues that stabilize the interface. Finally, these high-resolution structures have enabled researchers to explore novel allosteric binding sites. Recent improvements in computational chemistry have increased the accuracy of docking calculations, and with rapidly increasing computational power, researchers can explore the infinite chemical space and screen hundreds of millions of compounds. Structurally divergent compounds are likely to have unique biological properties that may potentially act as safer analgesics. Overall, the availability of high-resolution structures and a better understanding of the MOR structures may hold the key to successful opioid analogue discovery.

## Figures and Tables

**Table 1:** Four subtypes of opioid receptor and a list of their endogenous ligands.

Opioid Receptor Subtype	Endogenous Ligand
μ	β-endorphin, enkephalins, endomorphin-1, endomorphin-2
δ	β-endorphin, enkephalins
κ	dynorphin A, dynorphin B, α-neoendorphin
NOR ^1^	N/OFQ (nociceptin/orphanin FQ)

^1^ nociceptin/orphanin FQ opioid peptide receptor.

**Table 2:** Available structures of μ opioid receptor.

PDB ID	Ligand	Ligand Type	In Complex with	Structure Type	Resolution
4DKL	β-FNA	Antagonist	Lysozyme chimera	X-RAY	2.8
7UL4	Alvimopan	Antagonist	Megabody 6	Cryo-EM	2.8
5C1M	BU72	Full agonist	Nanobody 39	X-RAY	2.07
8EF5	Fentanyl	Full agonist	Gαi-1, Gβ-1, Gγ-2	Cryo-EM	3.3
8EF6	Morphine	Full agonist	Gαi-1, Gβ-1, Gγ-2, scFv16	Cryo-EM	3.2
6DDE	DAMGO	Full agonist	Gαi-1, Gβ-1, Gγ-2, scFv16	Cryo-EM	3.5
6DDF	DAMGO	Full agonist	Gαi-1, Gβ-1, Gγ-2	Cryo-EM	3.5
8EFQ	DAMGO	Full agonist	Gαi-1, Gβ-1, Gγ-2	Cryo-EM	3.3
7T2H	Lofentanil	Full agonist	Gαi-1, Gβ-1, Gγ-2, scFv16	Cryo-EM	3.2
7T2G	Mitragynine pseudoindoxyl	Full agonist	Gαi-1, Gβ-1, Gγ-2	Cryo-EM	2.5
8EFB	Oliceridine (TRV130)	Partial agonist	Gαi-1, Gβ-1, Gγ-2, scFv16	Cryo-EM	3.2
8EFL	SR17018	Partial agonist	Gαi-1, Gβ-1, Gγ-2, scFv16	Cryo-EM	3.2
8EFO	PZM21	Partial agonist	Gαi-1, Gβ-1, Gγ-2, scFv16	Cryo-EM	2.8
7SBF	PZM21	Partial agonist	Gαi-1, Gβ-1, Gγ-2, scFv16	Cryo-EM	2.9
7SCG	FH210	Partial agonist	Gαi-1, Gβ-1, Gγ-2, scFv16	Cryo-EM	3.0
7U2L	NNPPP ^1^	Bitopic ligand	Gαi-1, Gβ-1, Gγ-2, scFv16	Cryo-EM	3.2

^1^ N-(5-carbamimidamidopentyl)-N-[1-(2-phenylethyl) piperidin-4-yl] propanamide.

**Table 3:** The 2D structures of the ligands.

Ligand Type	2D Structure	
Agonist	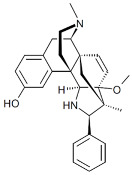 BU72	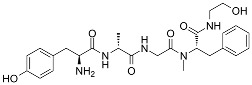 DAMGO
	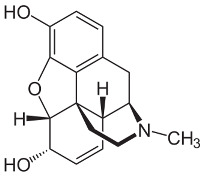 Morphine	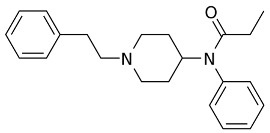 Fentanyl
	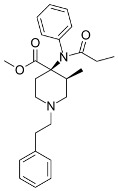 Lofentanil	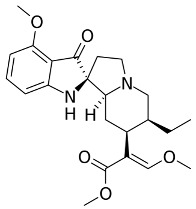 Mitragynine pseudoindoxyl
Partial agonist	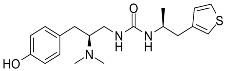 PZM21	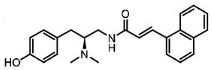 FH210
	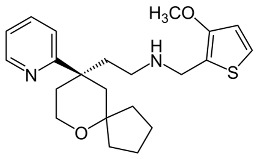 Oliceridine (TRV130)	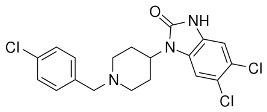 SR17018
Antagonist	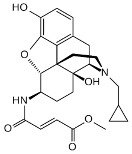 β-FNA	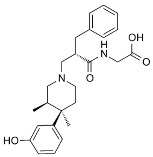 Alvimopan

**Table 4:** Binding pocket residue composition of each structure (residues within 5 Å of the ligand are included).

PDB ID	Ligand	Charged Residues	Polar Residues	Hydrophobic Residues
4DKL	β-FNA	5	2	8
7UL4	Alvimopan	3	6	11
5C1M	BU72	4	4	10
8EF5	Fentanyl	1	3	13
8EF6	Morphine	2	1	9
6DDE	DAMGO	4	5	10
6DDF6DDE	DAMGODAMGO	44	55	1010
6DDF8EFQ	DAMGO	24	55	1410
7T2H	Lofentanil	2	4	12
7T2G	Mitragynine pseudoindoxyl	3	5	10
8EFB	Oliceridine (TRV130)	1	3	15
8EFL	SR17018	1	4	13
8EFO	PZM21	2	4	13
7SBF	PZM21	3	5	11
7SCG	FH210	3	5	9

**Table 5:** Binding site solvent accessible surface area (SASA) and solvent accessible volume (SAV).

PDB ID	Ligand	SASA (Å^2^)	SAV (Å^3^)
4DKL	β-FNA	724	926
7UL4	Alvimopan	1016	1296
5C1M	BU72	1212	619
8EF5	Fentanyl	1062	1324
8EF6	Morphine	960	1097
6DDE	DAMGO	716	682
6DDF	DAMGO	631	588
8EFQ	DAMGO	827	938
7T2H	Lofentanil	821	1024
7T2G	Mitragynine pseudoindoxyl	612	674
8EFB	Oliceridine (TRV130)	1052	1067
8EFL	SR17018	1284	1875
8EFO	PZM21	949	989
7SBF	PZM21	1341	2126
7SCG	FH210	990	1330

## Data Availability

Not applicable.
